# Frailty Screening in the Emergency Department Enables Personalized Multidisciplinary Care for Geriatric Trauma Patients

**DOI:** 10.3390/jpm16020089

**Published:** 2026-02-04

**Authors:** Oluwafemi P. Owodunni, Tatsuya Norii, Sarah A. Moore, Sabrina L. Parks Bent, Ming-Li Wang, Cameron S. Crandall

**Affiliations:** 1 Department of Emergency Medicine, University of New Mexico Hospital, Albuquerque, NM 87131, USA; 2 Department of Surgery, University of New Mexico Hospital, Albuquerque, NM 87131, USA

**Keywords:** frailty, geriatric, trauma, risk assessment, age-friendly

## Abstract

Frailty is a multidomain reduction in physiologic reserve that impacts recovery and can contribute to poor outcomes following trauma beyond what chronological age, comorbidities, or injury severity predicts. In geriatric trauma patients, a large proportion are frail or prefrail on initial encounter in the emergency department, and because there are opportunities for actionable management plans, major trauma guidelines endorse systematic screening integrated into coordinated geriatric trauma care. We reviewed the literature and identified practical instruments used in the acute trauma setting for risk stratification. Additionally, we highlight the feasibility of using these instruments, as some can be completed via patient report, proxy input, or chart review when cognition, language, or caregiver availability limits history-taking. Implementation efforts succeed when shared mental models are leveraged and screening is embedded in the electronic health record system, linked to order sets and trigger-based pathways that offer downstream goal-directed care management, such as early mobility, delirium prevention, nutrition, medication review, and comprehensive geriatric assessment. Additionally, we highlight the importance of initiating early goals-of-care discussions and coordinating care with palliative care services. Resource-limited systems can preserve the same architecture by using nurse-led or allied staff-led screening, tele-geriatric consultation, and virtual interdisciplinary huddles. Lastly, we expand upon opportunities for longitudinal post-discharge follow-up. We describe how targeted initiatives translate research into practice, improve outcomes, and support longitudinal reassessment through in-person and telehealth follow-up visits.

## 1. Introduction

Frailty is a syndromic state of reduced physiologic capacity across multiple domains that cannot be inferred from age alone and should not be conflated with coexisting disease burden or baseline functional impairment [1,2,3,4,5]. Among geriatric patients with traumatic injury, frailty is common at presentation and is frequently associated with poor outcomes across the continuum of care [6,7]. Across prospective multicenter cohorts and administrative datasets, a large proportion of geriatric trauma patients meet frailty criteria, and another subset of these patients are prefrail [6,8]. Joseph et al. found that 14.7% of hospitalized trauma patients ≥70 years were frail at the index admission, and 12.9% were prefrail, predisposing patients to poor outcomes that were noted up to one year following the event [8]. Additionally, a large trauma-center cohort that assessed patients in the emergency department (ED) using the FRAIL index (evaluating risk based on: fatigue, resistance, ambulation, illnesses, and weight loss) reported comparably elevated prevalence of frailty, with a sizable proportion classified as frail or prefrail (67.2% combined) [9].

Frailty increases a patient’s risk stratum from the moment they arrive in the ED and persists well beyond the initial resuscitation. In ED-based cohorts, frail patients demonstrate substantially higher healthcare utilization and the need for more nuanced care coordination. Adeyemi et al. found an approximately three-fold increase in geriatric trauma team activations and inpatient admissions when compared to geriatric patients identified as nonfrail [9]. Discharge directly from the ED was notably uncommon in this group (38%), when compared to the patients admitted to the inpatient setting (62%). Following admission, frail patients were noted to have longer hospital lengths of stay (LOS), higher likelihood of in-hospital mortality, and increased need for post-acute placement [9]; this pattern is also noted across numerous studies [6,10,11,12]. Following discharge, poor frailty-associated outcomes persist; downstream events—including loss of functional independence, repeat falls, episodes of delirium, rehospitalization, transitions to residential care settings, and mortality—have been reported [8,13,14,15]. Falon et al. report that mortality at one-year post-discharge reached 28% vs. 5% in frail and nonfrail patients, respectively [13]. Functionally, the trajectory is also prolonged and unfavorable as frail patients are less likely to regain their pre-injury baseline [8,13,16]. Failure to rescue (FTR) in frail geriatric trauma patients is best framed as a systems-sensitive mortality phenotype, representing a key quality metric [11]. Frailty also emerges as the most informative predictor of FTR, with findings highlighting higher all-cause FTR rates in frail vs. nonfrail geriatric trauma patients, and as high as a four-fold increase in mortality irrespective of the mechanisms of injury [11,17]. The financial implications of frailty are also notable. Across inpatient, post-acute, and outpatient settings, frail patients generate substantially higher spending than nonfrail patients, with reported excess costs ranging from $616 to $32,549 and a pooled standardized mean difference of 0.55 (95% CI 0.44–0.67) [18]. In high-need subgroups with more complex chronic illness, Medicare expenditures were more than three-fold higher than in a healthier frail subgroup (cost ratio 3.05, 95% CI 2.48–3.75), with a markedly increased likelihood of a high-cost classification and greater use of nursing home care, inpatient hospitalizations, and ED visits [19].

While frailty is generally well-established in the literature, gaps still exist in the context of optimizing care for these patients, specifically by identifying high-risk patients through risk stratification and multidisciplinary collaborations for care coordination [20,21]. In this review, we identify frailty as a multidomain reduction in physiologic reserve that predisposes patients to poor outcomes. We then summarize how frailty screening has been deployed in acute trauma evaluation using practical and validated instruments and why it is important to incorporate screening and risk stratification into routine triage evaluation at first contact. We then highlight the opportunities for risk-informed multidisciplinary discussion and care coordination, beginning as soon as a patient is identified as frail. Importantly, when frail patients are not identified, teams implicitly accept uncontrolled bias in risk estimates, and inadvertently misclassify geriatric patients who are frail and operationally high-risk [20,21]. This position is supported by major trauma-surgery guidance, including the American College of Surgeons (ACS) *Best Practice Guidelines for Geriatric Trauma Management*, and parallel consensus work within the American Association for the Surgery of Trauma [12,22,23,24]. We conclude by highlighting the need to optimize care for frail patients, and for the next steps to focus on standardized screening tied to actionable pathways that enable individualized risk-appropriate care plans that should continue across care transitions.

## 2. Methods

We reviewed current geriatric trauma and age-friendly care guidelines to summarize consensus recommendations on frailty risk stratification, inpatient management, transitions of care, and implementation requirements relevant to geriatric trauma patients [24,25,26,27,28]. Furthermore, we identified systematic reviews and meta-analyses evaluating frailty in trauma populations by querying multiple electronic databases with structured combinations of MeSH headings, and free-text terms spanning trauma and injury descriptors (including traumatic injury, trauma), geriatric adult populations (older, elderly, aged), frailty constructs (frailty, frail, frailty index, frailty assessment, frailty status), and assessment language (assessment, screening, prediction, predictor, and risk). Eligible reviews were preferentially in the English language, and those registered in the International Prospective Register of Systematic Reviews and reported in accordance with the Preferred Reporting Items for Systematic Reviews and Meta-Analyses framework. Our query was conducted across Scopus, the Cochrane Library, MEDLINE/PubMed, Cumulative Index to Nursing and Allied Health Literature, EMBASE, and the Web of Science. The search window generally extended from 2000 to date, reflecting modern trauma care and frailty measurement practices.

The intent of this query was to identify the frailty risk assessment instruments that are used in trauma research. Using this approach, we identified eight eligible systematic reviews and meta-analyses that evaluated frailty in trauma patients, with five addressing trauma populations broadly [7,10,11,29,30], and three taking a more focused, domain-specific approach focused on particular injury patterns or trauma subtypes [31,32,33].

## 3. Validated Risk Stratification in Acute Trauma Settings

### 3.1. Overview

**Figure 1:**
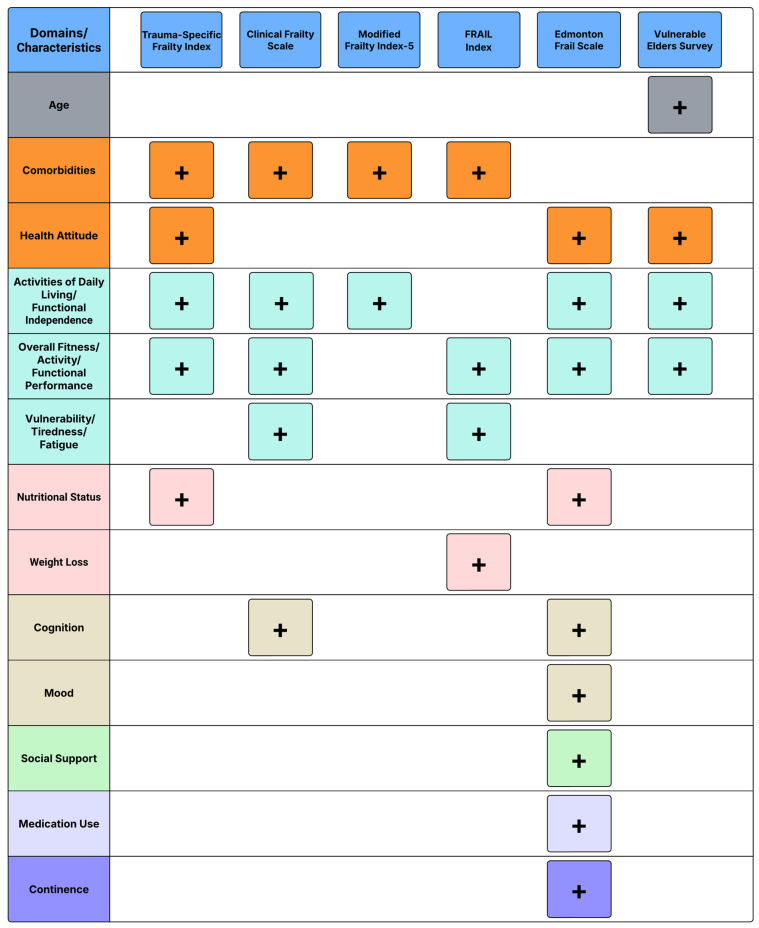
Domains and characteristics evaluated by different frailty risk stratification instruments. Plus indicates domain assessed by the risk assessment instrument.

Several frailty risk stratification instruments have been evaluated in geriatric trauma cohorts, and they differ in what they measure, how they are scored, and how easily they can be incorporated into workflows during trauma evaluation. The domains captured by the instruments (physiologic reserve, function, comorbidity burden, nutrition, and reported health attitudes) and the data requirements (history-only vs. structured variables vs. bedside judgment) determine whether screening can be performed reliably in the ED, and whether the result is available early enough to inform disposition, goals-of-care discussions, and discharge planning (Figure 1) [6,12,34,35,36].

Among trauma-oriented instruments, the Trauma-Specific Frailty Index (TSFI) has the strongest trauma-focused validation and is cited by the ACS as the preferred instrument for geriatric trauma assessment [6,12,24]. The TSFI uses 15 dichotomous items distributed across five domains (comorbidities, activities of daily living, reported health attitudes, functional status, and nutrition) (Figure 1). Scoring is contingent on the number of positive items divided by 15, and patients are stratified as nonfrail (TSFI ≤ 0.12), prefrail (TSFI 0.13–0.25), or frail (TSFI > 0.25) [6]. Operationally, its main advantage in the acute trauma setting is its feasibility, as it avoids performance-based testing such as gait speed or grip strength, which is often unrealistic or unsafe immediately after injury [6]. The Clinical Frailty Scale (CFS) takes a different approach to risk stratification. It is a nine-point, clinician-judgment scale ranging from very fit (1) to terminally ill (9) [35]. Because it relies on global clinical assessment rather than physical performance testing, it can be applied quickly in both the ED and inpatient trauma settings [35]. Its ease of deployment comes with subjectivity, so consistent application depends on shared mental models and local training [35]. The CFS is endorsed by the World Society of Emergency Surgery and the ACS as an acceptable alternative when TSFI is not practical [24,34].

Additionally, comorbidity-weighted indices remain common because they are easy to compute from routine documentation, especially in acute care settings. The Modified Frailty Index (mFI) and the abridged mFI-5 instrument emphasize chronic disease burden and functional deficits [36]. The mFI-5 uses five routinely captured conditions (congestive heart failure, chronic obstructive pulmonary disease, diabetes mellitus, hypertension, and an assessment for functional independence), making it advantageous during time-limited evaluations or when automated extraction from the electronic health record (EHR) system is desired [36]. Additionally, the FRAIL index offers another alternative with ease of deployment during ED evaluation or early hospitalization. It utilizes a five-item questionnaire that evaluates risk based on ambulation, fatigue, comorbidities, resistance, and weight loss [37]. Broader geriatric instruments, such as the Edmonton Frail Scale (EFS) [38,39], the Risk Analysis Index (RAI) [40,41], and the Vulnerable Elders Survey (VES-13) [42], also span multiple domains and are used widely in surgery. Their limitation in this context is their lack of ease of deployment and limited trauma-specific validation. However, instruments like the VES-13 and EFS, which are relatively streamlined compared to the RAI, can be deployed in the subacute trauma setting and are best viewed as adjuncts on a case-by-case basis (Figure 1) [39,43,44].

### 3.2. Barriers, Facilitators, and Workflow Integration

Identifying frail patients early during the initial trauma encounter changes what the team can do with the information. A documented frailty status supports risk-appropriate triage, appropriate disposition planning, and multidisciplinary planning that starts in the ED [6,10,12]. It also enables rapid integration of structured elder-care pathways, including the Hospital Elder Life Program (HELP) and Nurses Improving Care for Healthsystem Elders (NICHE), both of which have been associated with lower delirium rates, fewer inpatient complications, and reduced LOS in geriatric patients [12,45].

However, the routine implementation of frailty screening remains inconsistent because there are numerous barriers in the trauma workflow that impede implementation. These are usually high-acuity settings, and clinical data points may not be readily available, making risk assessment dependent solely on patient or caregiver recall [5,31]. Furthermore, performance-based measures are poorly suited to acute injury. Gait speed and grip strength are either unsafe, not feasible, or uninterpretable in patients with pain, immobilization, or an evolving physiology. While not a one-size-fits-all strategy, instrument heterogeneity and lack of a universally accepted instrument further introduce barriers to adoption, with variable calibration, domain coverage, and interrater reliability (Figure 1) [31]. Operationally, paper-based or free-text workflows predict low completion and poor downstream utilization. When embedded into the EHR system, completion improves with mandatory clinical decision support. The appropriate inclusion of screening instruments in order sets is advantageous as the frailty score and risk tier are captured and carried forward [46].

Furthermore, implementation succeeds when the pathways utilize validated frailty risk assessment instruments that can be deployed in real time. Providing brief, targeted staff training is advantageous for compliance. Standardized instructions promote consistent screening, thereby improving reliability across shifts and teams [12,46]. Consensus guidelines align on time and targeted strategies, e.g., screening should ideally be completed within 4 h of ED arrival, with a reference time comparison for baseline status from approximately 2–4 weeks prior to the index encounter [5,34,47]. Patients identified as high risk or frail should receive a comprehensive geriatric assessment (CGA) within 72 h of admission or after transfer from a critical care unit, when it can be executed reliably and translated into an interdisciplinary plan [34].

### 3.3. Considerations in Special Circumstances

For patients with baseline cognitive impairment or acute delirium, self-report is often unreliable. Proxy informants, including family members, long-term caregivers, or staff from assisted living, become the primary source for pre-injury function and day-to-day functional independence [31]. Instruments such as the TSFI and the FRAIL index lend themselves to this approach because they can be completed using proxy responses and, when needed, supplemented with chart review [6,9]. Proxy-based scoring is prone to errors in that the accuracy depends on how well the proxy understands the patient’s usual baseline. Additionally, it can be limited by the emotional and cognitive load of a traumatic event. Even with these limitations, programs report high practicality, with feasibility remaining strong [31]. Non-English-speaking patients require nuanced evaluation as ad hoc interpretation may undermine fidelity and produce systematic misclassification when information is obtained in the absence of qualified interpreter services [48,49]. The FRAIL index is often the preferred instrument due to its simplicity and validation in numerous languages, allowing rapid screening with fewer opportunities for semantic variation [9]. When no caregiver or proxy is available, the assessment should shift toward objective inputs. Baseline function and comorbidity burden can be gleaned from EHR and augmented with direct bedside evaluation when feasible. The TSFI can be operationalized with chart-derived elements when history is limited, and the mFI-5 is similarly practical because it relies on routinely documented comorbidities and functional dependence that are already captured in existing clinical records [50].

## 4. Frailty Pathways and Care Coordination

### 4.1. Lessons Learned from National Efforts

In the United States, care coordination for geriatric trauma patients is grounded in multidisciplinary, protocolized systems that extend beyond the index hospital encounter and continue through care transitions and follow-up [20,24,51]. The ACS *Best Practice Guidelines for Geriatric Trauma Management* highlight this strategy as an interprofessional process with a corresponding age-attuned care environment, e.g., delirium prevention, early mobilization, nutrition support, and sleep hygiene preservation as default standards of care [24]. These elements are designed to reduce preventable harm that disproportionately impacts frail patients during their hospital stay [20,21].

Within inpatient care, structured geriatric models have demonstrated measurable benefit when adapted to trauma. The Acute Care for Elders (ACE) model has been associated with shorter LOS, fewer non-home discharges, and lower costs [24]. Additionally, early integration of geriatric expertise has been shown to be associated with decreased delirium rates, reduced non-home discharges, fewer intensive care unit readmissions, and improved functional recovery at one year after injury [24]. The practical implication highlights substantial evidence supporting geriatric shared decision-making early enough to guide evaluation, optimization, and discharge planning [20,21,24,52]. Most well-established trauma programs operationalize this approach through a multidisciplinary team that is representative of trauma surgeons, geriatricians or hospitalists, nurses, physical and occupational therapists (PT/OT), pharmacists, social workers, and case managers [24]. Daily multidisciplinary rounds and standardized order sets are used to drive adherence, while checklists reduce omission errors [53]. Programs such as HELP and NICHE have demonstrated reductions in delirium and complications with accompanying reductions in LOS. These strategies reinforce the advantage of process redesign and how they can improve outcomes when consistently applied [53]. Geriatric trauma clinical pathways that incorporate CGA, frequent delirium screening, structured cognitive and functional evaluations, and care escalation algorithms have also been found to provide more consistent care delivery across teams [53].

Frailty risk stratification should trigger bundled, multidisciplinary frailty pathways. These pathways typically pair early ambulation with nonpharmacologic delirium-prevention strategies, standardized bowel and analgesia regimens, early nutrition and therapy consultation, and a CGA-based plan that aligns baseline function with realistic discharge goals [12]. Successful implementation efforts have shown promise in reducing delirium rates and 30-day readmissions [12,45]. Bryant et al. evaluated an interdisciplinary protocol for frail geriatric trauma patients and found a reduction in delirium rates from 21.6% to 12.5% (*p* = 0.04) and 30-day readmission from 9.6% to 2.7% (*p* = 0.01) with sustained reductions in multivariable analyses [45]. Additionally, transition of care is a high-risk interval for this population and requires deliberate design. The ACS guidelines emphasize post-acute care (PAC) rehabilitation as a key determinant of functional recovery and restoration of independence in activities of daily living [24]. The PAC planning generally spans skilled nursing facilities, inpatient rehabilitation, home health, and outpatient therapy, with these specialized teams assessing functional needs and setting individualized goals [24]. Transitional interventions that add structure, including nurse-led coordination, early home contact (including visits within 48 h of discharge), and reliable communication between in-hospital teams and outpatient primary care teams have been associated with lower readmission rates [54].

The Trauma Medical Home (TMH) model represents a more formalized collaboration across settings [55]. The TMH model incorporates an interdisciplinary team model to co-manage recovery with primary care teams led by a nurse care coordinator who performs a biweekly assessment with standardized instruments. The nurse coordinator serves as the communication hub, maintains individualized recovery plans, and initiates evidence-based protocols that address biopsychosocial domains (cognition, anxiety, depression, sleep, and physical function) [55]. The TMH model uses a combination of in-person and telehealth visits and incorporates weekly interdisciplinary meetings to review progress and revise plans. Zarzaur et al., in a multicenter randomized trial, found that this model favored improved mental health outcomes [55]. Outpatient coordination remains less uniform across systems, but increasingly follows a structured model of symptom monitoring, medication reconciliation, follow-up, and connection to community resources [55]. Rehabilitation is tailored to baseline and post-injury function through PT/OT, with speech–language pathology as indicated. The social work and case management teams address transportation, durable medical equipment, home modifications, and other social constraints that determine whether the discharge plan is executable [20,24]. High-performing programs connect inpatient, transitional, and outpatient work into a single continuum using standardized protocols, multidisciplinary case reviews, shared electronic records, and proactive communication strategies [25,55].

The John A. Hartford Foundation and the Institute for Healthcare Improvement developed the initiative for the Age-Friendly Health Systems (AFHS) 4M framework (medication, mentation, mobility, and what matters) [25]. This initiative provides a usable structure for implementation across settings, keeping care aligned with frailty-related risks, and centering decisions on the patient’s priorities [25,56]. In 2025, the Centers for Medicare & Medicaid Services (CMS) introduced the AFHS measure. This measure requires hospitals to report structured evidence of age-friendly practices across five attestation domains aligned with the 4Ms, and include identifying health care goals, medication reconciliation, frailty screening and intervention (including mentation, mobility, and nutrition), social vulnerability, and age-friendly care leadership [27]. Under CMS guidance, hospitals are required to report the measure to receive full Medicare reimbursement; nonreporting is penalized. This policy shift created an opportunity to incorporate the AFHS framework as required infrastructure and allows hospitals to operationalize frailty screening and geriatric risk management as standard patient-centered care rather than discretionary quality efforts [26,27]. Voluntary quality improvement initiatives alone rarely produce durable frailty screening at scale because they compete with throughput pressure, variable staffing, and inconsistent ownership. Adoption may change with institutional priorities. In practice, incentives advance adoption by aligning three main domains beyond voluntary efforts alone. First, they mandate resource allocation for staffing and training (nurse champions, transitional care coverage, geriatrics consult pathways, and telehealth infrastructure). Second, they force integration into the EHR, which is the only scalable mechanism for consistent screening, automated triggers, and pathway activation. Third, they create feedback loops through required reporting, allowing systems to detect defects, benchmark performance, and justify iterative redesign [57,58,59].

Furthermore, the ACS *Trauma Quality Improvement Program—Palliative Care Best Practices Guidelines* provide structured guidance for care coordination for the geriatric adult trauma patient with the palliative care team [28]. The guidelines center on a structured palliative care assessment and triage processes that begin on admission and extend through the first 72 h [28]. Patients who screen positive commonly have uncertain survival or functional recovery based on an initial risk assessment, which is contingent on a combination of factors (injury severity, frailty, comorbidities, and age). A positive screen is intended to trigger early advance care planning and a formal goals-of-care (GOC) discussion [28]. The implementation of this guideline is framed as a performance measure that requires buy-in from leadership, targeted training in clear, patient-centered communication, and EHR-integrated screening triggers. Additionally, adherence is meant to be tracked with structured process and outcome surveillance, which evaluates early advance directive identification, timeliness of GOC conversations (within 72 h), consultation rates, protocol availability, and the reviews of end-of-life care [28,60].

In the ED, there are ongoing efforts supporting Geriatric Emergency Department Accreditation (GEDA). These efforts were developed by the American College of Emergency Physicians in collaboration with the American Geriatrics Society and partner organizations (including the Society for Academic Emergency Medicine and the Emergency Nurses Association) to standardize leadership, education, care processes, transitions, and the physical environment for geriatric patients [61,62]. The deliverable is an ED that can demonstrate reliable screening and protocolized responses to geriatric syndromes with structured surveillance and reporting. Recent updates and parallel national efforts are pulling GEDA toward close alignment with the AFHS 4Ms framework [61,62]. The updated Geriatric Emergency Department Guidelines 2.0 initiative is expanding its recommendations to address delirium, dementia, falls, frailty, medication reconciliation, palliative care, and elder abuse [63]. Moreover, the feasibility of implementation across diverse resource settings is focused on broader uptake of the core elements, while preserving the need to adapt staffing models, screening workflows, and referral pathways to local capacity [61,62,63].

### 4.2. Lessons Learned from International Efforts

Outside the United States, geriatric trauma coordination is shaped by national financing structures, workforce availability, and geography so there is wide variation contingent on these factors. In the United Kingdom, major trauma centers have moved toward earlier, nurse-led frailty identification in the ED, related to National Health Service England commissioning incentives that explicitly support frailty-specific trauma pathways [64]. Even with that policy leverage, delivery is inconsistent at the hospital level. Acute pathways for geriatric trauma patients differ substantially across sites, and geriatrician review within 72 h is not reliably achieved. Factors like age, staffing, and local service capacity are key determinants of care coordination [65]. One response to address barriers in post-discharge follow-up was the implementation of a structured follow-up model (e.g., the Silver Trauma Review Clinic), which provides a multidisciplinary review for geriatric trauma patients with non-operative injuries that pairs CGA with a tertiary survey for missed injuries, bone health evaluation and fall-risk assessment, cognitive screening, and targeted PT/OT referral [66]. The data from trials of frailty-oriented case management in community settings, typically led by health or social care professionals with multidisciplinary support, displayed limited incremental benefit over usual care for major outcomes, with little or no difference observed in mortality, non-home discharge, functional independence, hospital admission, or costs [67]. The operational takeaway is that outcomes depend on what the coordinator can actually change (services, timing, accountability, and follow-through), and not only on the presence or involvement of a coordinator.

In Canada, targeted-frailty care coordination is more explicitly focused on consensus guidance for patient-centered frailty management with early detection and interdisciplinary input [68,69]. Furthermore, in Australia, the geography impacts the pathway design and implementation efforts. Low population density and long transport distances between injury location and definitive care settings impose structural barriers on what can be centralized and how quickly geriatric expertise can be delivered. National geriatric trauma guidelines are not currently established, but trauma clinicians have converged on priority elements for future standards [70]. Qualitative efforts contribute to the perspective on recovery, and identify recurring cross-sector defect-prone areas, highlighting the practical need for advocacy to coordinate care across healthcare and social-support systems [71].

### 4.3. Patient-Centered Outcome Assessment

Patient-centered outcome reporting in coordinated geriatric trauma pathways has relied on established instruments that capture symptoms, function, and health-related quality of life. Measures commonly used include the 36-Item Short-Form Health Survey (SF-36); EuroQol 5-Dimension 5-Levels ([EQ-5D-5L] for quality of life) [72]; the Short Physical Performance Battery (for physical performance) [73]; Patient Health Questionnaire-9 (PHQ-9); Generalized Anxiety Disorder-7 (GAD-7) (for depressive and anxiety symptoms) [55]; and the Katz Index of Independence in Activities of Daily Living scale and Lawton–Brody Instrumental Activities of Daily Living scale (to quantify independence with performing activities) [73]. Care coordination studies evaluating caregiver experience are limited. However, the Family Satisfaction with End-of-Life Care scale has been previously used to obtain caregivers’ perspectives [74]. Overall, the findings are mixed as estimates for patient satisfaction, engagement, and overall quality-of-life outcomes tend to be marginal, even when process measures improve [55]. The TMH model adds an important perspective to a patient’s trajectory, as collaborative care appears to matter most for recovery when a patient has a high baseline psychosocial burden. It was noted that high baseline anxiety or depressive symptoms demonstrated improvement in the mental health component of quality of life when assigned to TMH, with corresponding favorable improvements in SF-36, GAD-7, and PHQ-9 metrics [55].

### 4.4. Real-World Implementation and Best Practices

**Figure 2:**
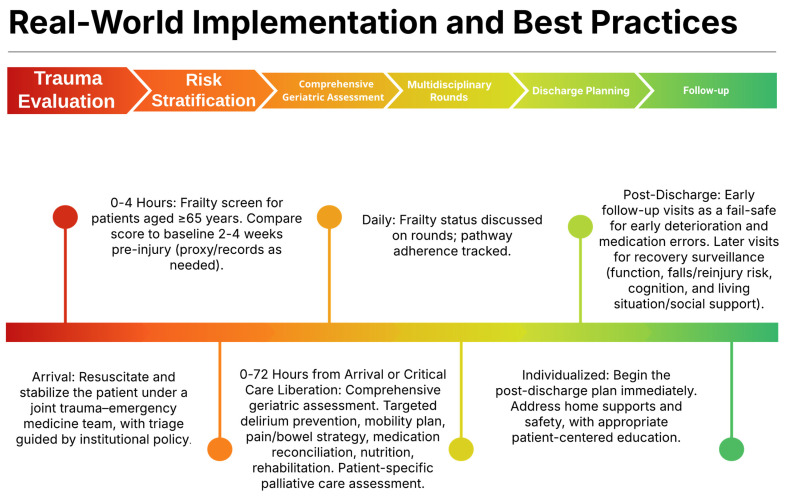
Guideline-aligned comprehensive geriatric trauma pathway.

Implementation efforts depend on local institutional capacity and should be tailored to the site-specific workforce and workflow. The ACS *Best Practice Guidelines for Geriatric Trauma Management* explicitly acknowledge this reality, positioning the primary care team at the center for care coordination and endorsing remote consultative geriatrics models when on-site expertise is unavailable [24]. Frailty screening should function as the earliest identification mechanism, and not a downstream specialty task. Clinician- or allied-staff-administered instruments allow high-risk identification without waiting for performance testing or specialty evaluation, preserving the window to identify, stratify, and coordinate care (Figure 2) [24]. Once risk is defined, protocolized frailty pathways inform practice even in limited resource settings, as standardized bundles are initiated by default, limiting the influence of individual clinician preference (Figure 2) [12,45].

Collaborative care models provide an actionable framework for longitudinal management when limited by location or capacity (Figure 2). The TMH approach operationalizes this effort by leveraging the skills of a nurse care coordinator [55]. Weekly interdisciplinary reviews do not require a shared physical space as virtual team meetings can deliver the same function, and the discussion can be structured to follow the patient’s course, identify barriers early, and preemptively provide revised individualized plans. Additionally, on a system level, low-cost environmental design modifications can be advantageous and offer a high-yield return on investment. For example, geriatric-friendly modifications (installation of handrails, clutter control, using contrast-enhancing paint colors, prominent clocks/calendars, and flexible visiting policies that support orientation) targeting delirium and anxiety risk can be deployed incrementally [24]. The HELP and NICHE initiatives offer pragmatic protocol content that can be scaled and adapted to resource-limited settings, with the emphasis on reproducible core interventions [24,53]. The same logic applies to workflow, as order sets, automated triggers for geriatric trauma admission processes, daily multidisciplinary rounding structures, and checklists for delirium prevention and functional assessment can be embedded into routine documentation so that reliability does not depend on memory or individual enthusiasm [53]. Education should be brief, multidisciplinary, and reinforced by local champions in each discipline, with patient- and family-facing materials that explain the plan in plain language to reduce miscommunication during transitions (Figure 2) [53].

Transitions are a potential area of concern, especially in resource-limited settings where post-acute resources may be scarce or inconsistently available [24]. Transitional care, therefore, needs a stripped-down, executable design focusing on rehabilitation planning led by the clinicians, supplemented by telehealth consultation when expertise gaps are obvious, early home contact when feasible, and closed-loop communication with the primary care team [24,55]. Nurse- or social-worker-led case management is often the most realistic coordination mechanism in resource-limited settings, but effectiveness depends on the integration of a CGA element, individualized plans, and scheduled reassessment delivered through a structured model [67].

Finally, resource-limited implementation fails when social needs are not prioritized. Care coordination should incorporate structured screening for social determinants during intake and explicitly evaluate individual needs for community resources because transportation, caregiving capacity, food insecurity, housing stability, and cultural context frequently determine whether the discharge plan is feasible [24]. Guidance from the ACS supports early appraisal of the geriatric patient’s support structure, including psychological needs and cultural or spiritual identity and strengths that may support recovery [24]. The TMH model similarly incorporates recurrent assessment of social needs and functional barriers, and links these high-risk patients to the appropriate resources, with coordinators serving as facilitators [55]. Lastly, transitional care frameworks can be adapted to align social-needs screening with established social determinants of health domains, thereby reducing preventable adverse events and mitigating disparities during recovery [49,75].

## 5. Limitations

A key limitation of this review is that geriatric trauma pathways are supported primarily by pragmatic best-practice guidance, single-center protocols, and heterogeneous observational data, while rigorous implementation science remains underdeveloped; there are few effectiveness–implementation hybrid trials, limited dissemination and scale-up evaluations, and limited reporting on adoption, fidelity, and sustainability. Therefore, this review summarizes the available practice evidence while highlighting the need for methodologically robust studies that test how these pathways can be implemented reliably across diverse trauma settings.

Furthermore, this review was not designed as a full scoping review with exhaustive capture of the literature, nor a multi-reviewer independent screening. The frailty instruments compared may be prone to selection bias driven by which reviews were identified and which studies those reviews include. Nonetheless, the instruments highlighted in the current review reflect what has been reported in trauma research and support the face validity that these instruments reflect current clinical practice despite the methodological limitations. Additionally, patient-reported outcomes and patient-defined priorities are underrepresented in the evidence synthesized here, leaving an incomplete view of recovery from the standpoint of frail geriatric survivors and their caregivers. Future implementation work should incorporate qualitative perspectives that capture feasibility, tradeoffs, and failure points during recovery [76]. Additionally, future qualitative studies should integrate shared decision-making frameworks so pathways reflect both survivor goals and caregiver capacity.

## 6. Conclusions

Frailty contributes to a predictable cascade of delirium, immobility, institutionalization, mortality, and other poor outcomes for geriatric patients following an acute traumatic event, unless the system intervenes early. High-performing programs treat frailty as an admission vital sign, incorporate risk stratification into the EHR, utilize standardized multidisciplinary pathways that promote targeted patient optimization plans, and include discharge planning. Resource constraints should not negate this approach; rather, we can incorporate feasible screening, protocolized response, and leverage local workflows to coordinate care. Future research should evaluate scalable frailty pathways that pair digital health and integrated care models with large language models to extract pre-injury baseline function and patient priorities from unstructured records in near real time, reducing clinician workload while triggering consistent, audit-ready interventions across settings [77,78].

## Data Availability

No new data were created or analyzed in this study. Data sharing is not applicable to this article.
